# Loss of Response Gene to Complement 32 (RGC-32) in Diabetic Mouse Retina Is Involved in Retinopathy Development

**DOI:** 10.3390/ijms19113629

**Published:** 2018-11-17

**Authors:** Wen-Ling Liao, Jane-Ming Lin, Shih-Ping Liu, Shih-Yin Chen, Hui-Ju Lin, Yeh-Han Wang, Yu-Jie Lei, Yu-Chuen Huang, Fuu-Jen Tsai

**Affiliations:** 1 Center for Personalized Medicine, China Medical University Hospital and Graduate Institute of Integrated Medicine, China Medical University, Taichung 404, Taiwan; wl0129@mail.cmu.edu.tw; 2 School of Chinese Medicine, China Medical University, Taichung 404, Taiwan; d4301@seed.net.tw (J.-M.L.); chenshihy@mail.cmu.edu.tw (S.-Y.C.); irisluu2396@gmail.com (H.-J.L.); 3 Department of Ophthalmology, China Medical University Hospital, Taichung 404, Taiwan; 4 Center for Translational Medicine, China Medical University Hospital and Graduate Institute of Biomedical Science, China Medical University, Taichung 404, Taiwan and Department of Social Work, Asia University, Taichung 413, Taiwan; spliu@mail.cmu.edu.tw; 5 Department of Medical Research, China Medical University Hospital, Taichung 404, Taiwan; yjlei0824@gmail.com; 6 Department of Anatomical Pathology, Taipei Institute of Pathology, Taipei 103, Taiwan and Institute of Public Health, National Yang-Ming University, Taipei 112, Taiwan; yehanwang@gmail.com; 7 Department of Medical Genetics, China Medical University Hospital and Children’s Hospital of China Medical University, Taichung 404, Taiwan

**Keywords:** RGC-32, T2D, diabetic retinopathy, photoreceptor, apoptosis

## Abstract

Diabetic retinopathy (DR) is a severe and recurrent microvascular complication in diabetes. The multifunctional response gene to complement 32 (RGC-32) is involved in the regulation of cell cycle, proliferation, and apoptosis. To investigate the role of RGC-32 in the development of DR, we used human retinal microvascular endothelial cells under high-glucose conditions and type 2 diabetes (T2D) mice (*+Lepr^db^/ + Lepr^db^, db/db*). The results showed that RGC-32 expression increased moderately in human retinal endothelial cells under hyperglycemic conditions. Histopathology and RGC-32 expression showed no significant changes between T2D and control mice retina at 16 and 24 weeks of age. However, RGC-32 expression was significantly decreased in T2D mouse retina compared to the control group at 32 weeks of age, which develop features of the early clinical stages of DR, namely reduced retinal thickness and increased ganglion cell death. Moreover, immunohistochemistry showed that RGC-32 was predominantly expressed in the photoreceptor inner segments of control mice, while the expression was dramatically lowered in the T2D retinas. Furthermore, we found that the level of anti-apoptotic protein Bcl-2 was decreased (approximately 2-fold) with a concomitant increase in cleaved caspase-3 (approximately 3-fold) in T2D retina compared to control. In summary, RGC-32 may lose its expression in T2D retina with features of DR, suggesting that it plays a critical role in DR pathogenesis.

## 1. Introduction

Diabetic retinopathy (DR) is a common complication in diabetic patients due to abnormalities in the retinal neurovascular structure and is a major cause of vision impairment worldwide [1,2,3]. Risk factors in the development of DR include poor glycemic control, longer duration of diabetes, hypertension, hyperlipidemia, and albuminuria [4,5,6,7,8,9]. 

Response gene to complement 32 (*RGC-32*) is located on chromosome 13q14.11 and is expressed in numerous organs and tissues [10]. RGC-32 is involved in the regulation of cell proliferation, apoptosis, differentiation [11,12,13,14,15], and tumorigenesis [16,17,18]. RGC-32 expression varies among cancer subtypes and is either up- or down-regulated based on disease context [19]. Previously, An et al., in 2009, reported that RGC-32 expression was increased under hypoxia via hypoxia inducible factor 1α/vascular endothelial growth factor (VEGF) induction [20]. Overexpression of RGC-32 reduced the proliferation and migration of endothelial cells and destabilized vascular structure formation in vitro [20]. In addition, RGC-32 plays an important role in the regulation of glucose homeostasis and lipid metabolism and in the development of obesity and insulin resistance [21,22,23]. Irregular RGC-32 expression is suggested to cause obesity, insulin resistance, and endothelial dysfunction, all of which may play an important role in the pathogenesis of diabetes and the ensuing complications.

Nevertheless, it is unclear if RGC-32 plays a role in DR pathogenesis. Therefore, we investigated RGC-32 expression in human retinal cells under hyperglycemic and normal conditions. In addition, we used the type 2 diabetes (T2D) mouse model at 16, 24, and 32 weeks of age to investigate the role of RGC-32 in mouse retina.

## 2. Results

### 2.1. RGC-32 Expression in Human Retinal Cells under Hyperglycemic Condition

**Figure 1:**
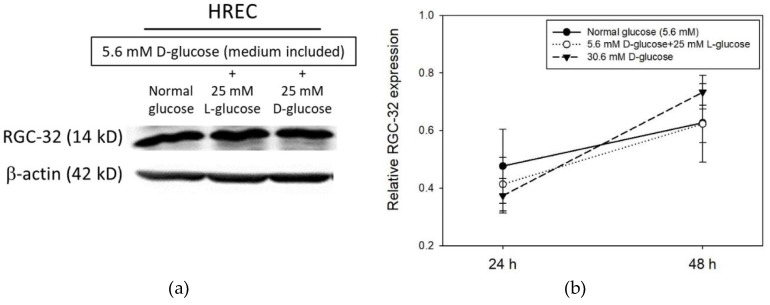
(**a**) Representative western blot image of RGC-32 expression in HREC under high glucose condition for 24 h (**b**) RGC-32 expression relative to that of β-actin in retinal cell under high glucose condition for 24 and 48 h. Data are presented as mean ± SD and differences between means were compared by ANOVA test.

We investigated the changes in RGC-32 expression in human retinal endothelial cells (HRECs) under normal and hyperglycemic conditions (Figure 1a). HRECs were treated with normal (5.6 mM) or high (5.6 mM + 25 mM) concentrations of d-glucose for 24 h or 48 h. Cells were also exposed to 25 mM l-glucose + 5 mM d-glucose as an osmotic control for the experiments. Western blot results showed an increase in RGC-32 protein level from 24 to 48 h in HRECs under both normal and high glucose concentration conditions (Figure 1b). However, the level of RGC-32 expression in HRECs compared to the normal glucose condition was moderately decreased at 24 h but was moderately increased in HRECs at 48 h under high concentration of d-glucose (Figure 1b). 

### 2.2. Histopathology in T2D Mouse Retina

**Figure 2:**
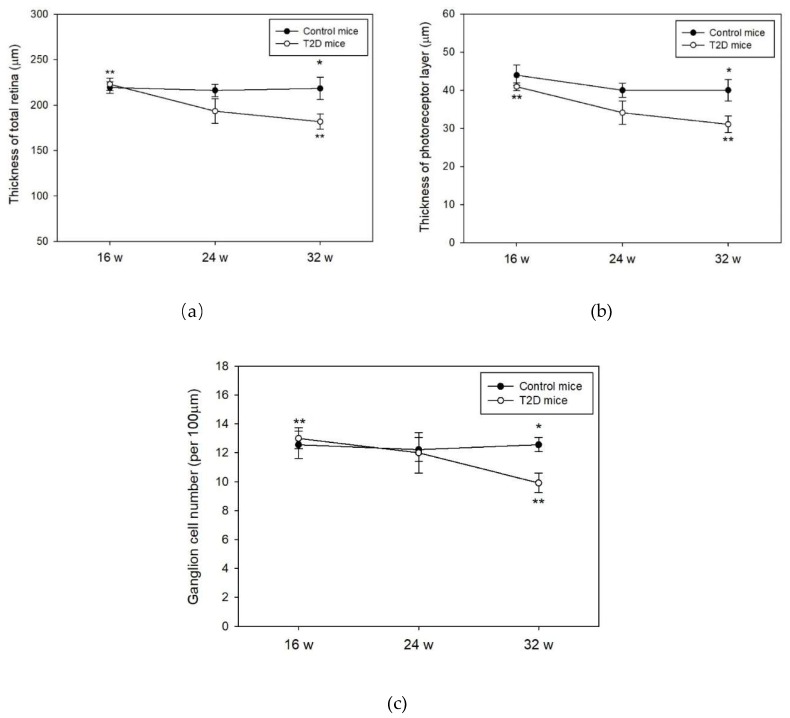
Histological evaluation of the mice retina at 16, 24, and 32 weeks (w) of age. Data are presented as mean ± SD. (**a**) Thickness of total retina, * *p* = 0.032 (*t*-test), ** *p* = 0.010 (T2D mice between 16 and 32 weeks of age, Kruskal-Wallis test followed by Dunn’s test); and (**b**) Thickness of photoreceptors, * *p* = 0.028 (*t*-test), ** *p* = 0.004 (T2D mice between 16 and 32 weeks of age, Kruskal-Wallis test followed by Dunn’s test) were measured in T2D and control mice retina; (**c**) Ganglion cell numbers were counted in T2D and control mice retina, * *p* = 0.003 (*t*-test), ** *p* = 0.023 (T2D mice between 16 and 32 weeks of age, Kruskal-Wallis test followed by Dunn’s test).

Histological evaluation of the retinas was performed on 16, 24, and 32-week-old mice. No significant changes in the thickness of the total retina, photoreceptor, and the number of the retinal ganglion cells were observed between T2D and control mice at 16 and 24 weeks of age (Figure 2a–c). At age 32 weeks, we observed significantly lowered thickness of the total retina and the photoreceptor layers in T2D mice compared to control (total retinal thickness: T2D vs. control mice: 181.8 ± 8.3 μm vs. 218.3 ± 12.2 μm, *p* = 0.032, Figure 2a; photoreceptor layer: T2D vs. control mice: 40.0 ± 2.8 μm vs. 31.1 ± 2.1 μm, *p* = 0.028, Figure 2b). The number of retinal ganglion cells in T2D mice was also significantly reduced compared to control mice at 32 weeks of age (T2D vs. control mice: 9.9 ± 2.4 cells/100 μm vs. 12.6 ± 2.0 cells/100 μm, *p* = 0.003, Figure 2c). Furthermore, the thickness of the total retina and photoreceptor layer as well as the number of retinal ganglion cells revealed a time dependent reduction in the T2D mice. The reduced retinal thickness and lowered number of ganglion cells in T2D mice are consistent with the features of the early clinical stages of DR. We, therefore, used the 32-week-old T2D mice to mimic the pathogenesis of clinical DR in this study.

### 2.3. Time-Dependent Change in RGC-32 Expression in T2D Mouse Retina

**Figure 3:**
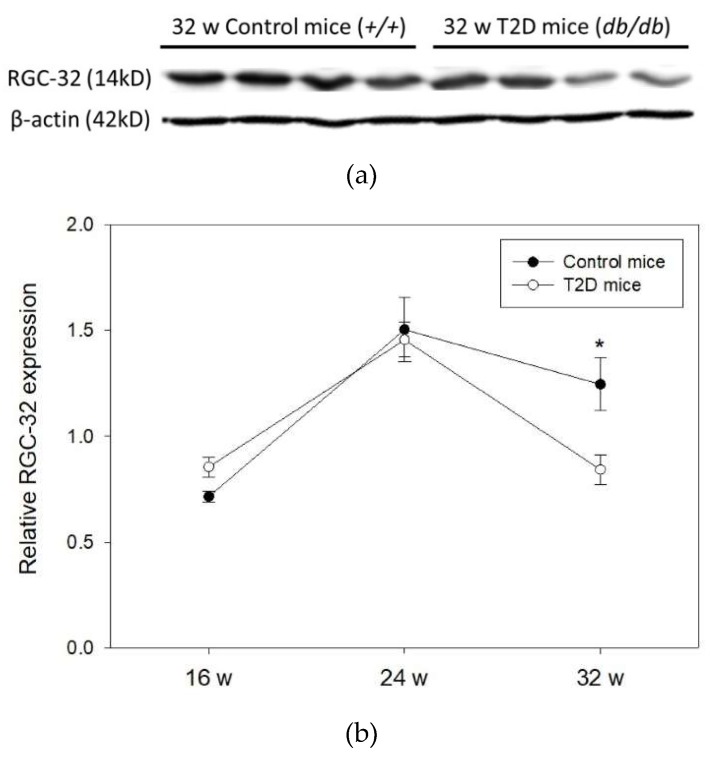
(**a**) Representative western blot image of RGC-32 expression in T2D and control mouse retina (**b**) RGC-32 expression relative to that of β-actin in mouse retina at 16, 24, and 32 weeks (w) of age. Data are presented as mean ± SD. * *p* = 0.030 (*t*-test).

**Figure 4:**
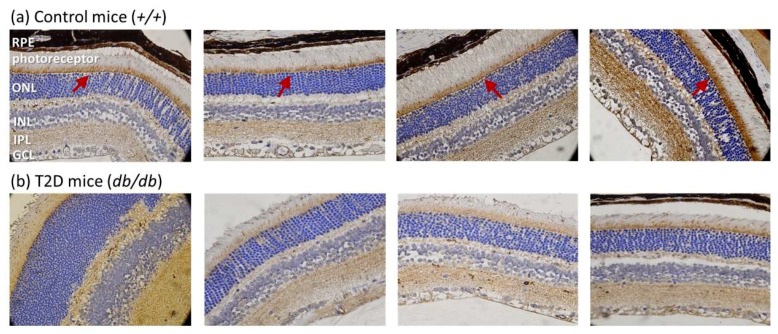
Representative images of immunohistochemical staining of RGC-32 expression in mouse retina at 32 weeks of age (magnification = 400×) (**a**) RGC-32 is prominently expressed in the photoreceptor inner segments in control mice (arrow); (**b**) RGC-32 expression is decreased in T2D mouse retina.

Next, we evaluated RGC-32 expression in the retina of 16, 24, and 32-week-old mice (Figure 3a). Western blots showed that RGC-32 levels were increased from 16 to 24 weeks of age, but were decreased from 24 to 32 weeks of age either in T2D or control mice. No significant changes were observed between T2D and control mice at 16 and 24 weeks of age. Nevertheless, RGC-32 levels were significantly decreased in T2D mice retina compared to control mice at 32 weeks (relative RGC-32 expression: T2D mice, 0.84 ± 0.07 vs. control mice, 1.25 ± 0.12; *p* = 0.030, Figure 3b). Further results obtained from immunohistochemical staining showed that RGC-32 expression decreased in T2D mouse retina compared to control mice at 32 weeks of age (Figure 4a,b). The localization of RGC-32 expression was prominent in the photoreceptor inner segments (indicated by arrows in Figure 4a). 

### 2.4. Increased Apoptosis of Retinal Cells in T2D Mice at 32 Weeks of Age

**Figure 5:**
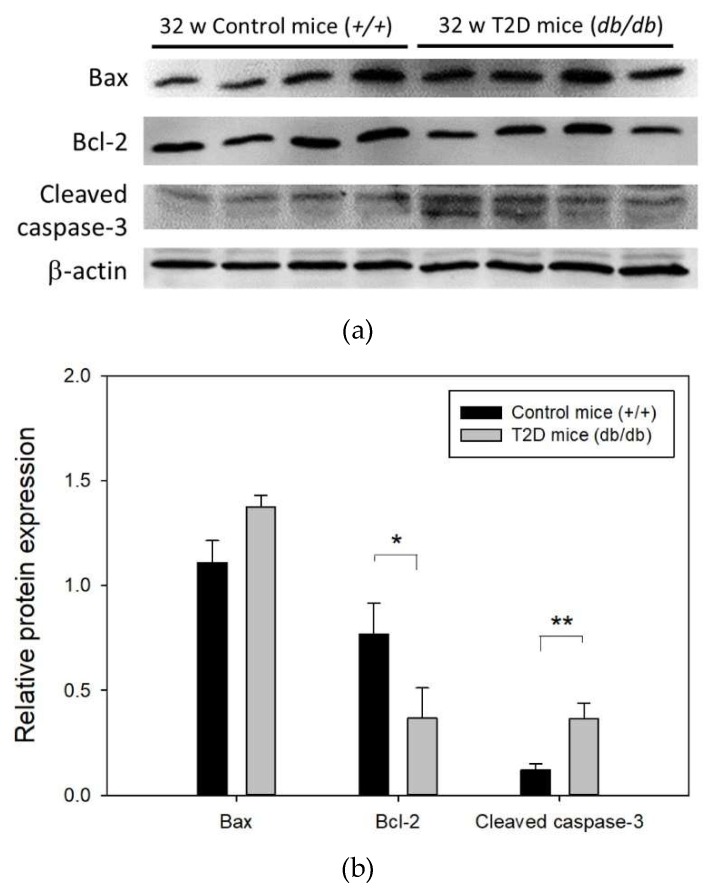
(**a**) Representative western blot image of apoptosis related protein expression in T2D and control mouse retina (**b**) Results of Bax, Bcl-2, and cleaved caspase-3 expression in mouse retina normalized to that of the internal control, β-actin. Data are presented as mean ± SD. * *p* = 0.008, ** *p* = 0.025 (*t*-test). w: weeks.

We next investigated the levels of apoptosis-related proteins Bax, Bcl-2, and cleaved caspase-3 in the mouse retina at 32 weeks of age (Figure 5a). Western blot results showed that compared to control mice, the level of pro-apoptotic protein Bax was increased (relative Bax expression: T2D mice, 1.11 ± 0.10 vs. control mice, 1.38 ± 0.05; *p* = 0.064), that of anti-apoptotic protein Bcl-2 was reduced approximately 2-fold (relative Bcl-2 expression: T2D mice, 0.77 ± 0.07 vs. control mice, 0.37 ± 0.07; *p* = 0.008), and that of cleaved caspase-3 was significantly increased by approximately 3-fold (relative cleaved caspase-3 expression: T2D mice, 0.12 ± 0.03 vs. control mice, 0.36 ± 0.08; *p* = 0.025) in T2D mouse retina (Figure 5b). These results suggest increased apoptosis of retinal cells in T2D mice at 32 weeks of age.

## 3. Discussion:

To the best of our knowledge, we are the first group to report reduced RGC-32 expression in T2D mouse retina, particularly in the photoreceptor inner segments and to suggest its potential role in retinopathy development. RGC-32, also known as regulator of cell cycle (RGCC), is primarily a cell-cycle regulator and is involved in cell proliferation, apoptosis, differentiation [11,12,13,14,15], and tumorigenesis [16,17,18]. Apoptosis is one of the key diabetes-induced cell death pathways in multiple retinal cell types and is believed to be a crucial step in DR pathogenesis [24]. Knockdown of RGC-32 has been suggested to inhibit cell growth and invasion and promote spontaneous apoptosis in lung cancer cells [15]. We observed loss of RGC-32 expression in T2D mouse retina at 32 weeks of age and decreased expression of the anti-apoptosis protein Bcl-2 with a concomitant increase in cleaved caspase-3, which plays a central role in the execution-phase of cell apoptosis. However, as it cannot be clarified whether this phenomenon of apoptosis in T2D mice retina is due to RGC-32 reduction, as diabetes-induced cell death may be the likely cause. Further study is needed to determine whether knockdown of RGC-32 induces retinal cell apoptosis.

Photoreceptors are specialized light-sensing cells unique to the retina and capable of visual phototransduction. Previous studies suggested that diabetes-induced structural and functional alterations in photoreceptors may play a role in DR pathogenesis (reviewed in [25]). We not only observed significant thinning of the total retinal layer but also a reduced thickness of the photoreceptor layer in T2D mice compared to control. Such retinal abnormalities were also reported in other studies in *db/db* mice over 8–24 weeks of diabetes [26,27]. Additionally, RGC-32 in the control retina was predominantly expressed in the photoreceptor inner segments that contain abundant mitochondria that play a key role in activating intrinsic apoptosis in mammalian cells [28]. Colocalization studies of RGC-32 with mitochondria specific proteins may reveal greater information in the future. Since photoreceptors may play an important role in diabetic-induced degeneration of the retinal capillaries [25], loss of RGC-32 expression in T2D mouse retina photoreceptor should be further investigated to elucidate the mechanism of DR pathogenesis.

A previous report indicated that RGC-32 expression can be induced by high-glucose conditions in human microvascular endothelial cells [22]. We observed a moderate increase in RGC-32 levels in HRECs under high glucose for 48 h. It is interesting to note that the level of RGC-32 expression increased between 24 and 48 h in normal or high glucose conditions. We believe this may be dependent on cell proliferation or regulation events. RGC-32 expression in mice retina increased before 24 weeks of age but decreased at 32 weeks, dramatically reducing in T2D mice retina compared to control. It is not surprising that RGC-32 expression in T2D mice starkly contrasted the observations in vitro high-glucose treatments, underlining the fact that high-glucose conditions in retinal 2D cell culture models may not reproduce the DR microenvironment of the T2D retina. 

Furthermore, RGC-32 expression has been reported to be induced in the adipose tissue of high-fat diet-induced obese mice [21,22]. Yet, the expression of RGC-32 is reversed in *db/db* mouse retina, which is a T2D mice model with spontaneous mutation in the leptin receptor. The contrasting RGC-32 expression pattern in *db/db* mouse retina and high-fat diet-induced obese mice adipose tissue may be due to differences in tissue or in the experimental mice models. Even though obesity is an important T2D risk factor and high-fat diet-induced obesity mice share several phenotypic features (e.g., obesity, increased glucose intolerance, insulin resistance, and elevated glucose levels) with *db/db* mice, the high-fat diet-induced obese mice do not typically develop basal hyperglycemia. Nevertheless, this suggests that RGC-32 may play different roles specific to the different stages of diabetes.

In summary, RGC-32 may play a role in DR development and is a potential drug target for future DR therapeutic strategies.

## 4. Materials and Methods

### 4.1. Cell Culture

We used human retinal microvascular endothelial cells (HREC) purchased from Cell Biologics company (Cell Biologics, Inc., Chicago, IL, USA) for our in vitro experiments. HRECs were maintained in tissue culture flasks pre-coated with a gelatin-based solution for 10 min and incubated in complete human endothelial medium (with 5.6 mM d-Glucose containing essential and non-essential amino acids, vitamins, organic and inorganic compounds, hormones, growth factors, trace minerals) supplemented with endothelial cell growth supplement (containing VEGF, heparin, epidermal growth factor, fibroblast growth factor, hydrocortisone, and l-Glutamine), antibiotics, and 10% of fetal bovine serum (Cell Biologics, Inc, Chicago, IL, USA). All cells were maintained at 37 °C in a humidified incubator with 5% CO_2_. For experiments involving the effects of normal or high glucose conditions, cells were incubated with 5.6 mM (medium included), 25 mM d-Glucose (additional added, final concentration 30.6 mM) or 25 mM l-Glucose (medium includes 5.6 mM d-Glucose + additional 25 mM l-Glucose) for 24 and 48 h after cell were seeded. l-Glucose treatment was used as an osmotic control for experiments. Each set of experiments was performed three times independently.

### 4.2. Type 2 Diabetes (T2D) Mouse Model 

Six-week-old male BKS.Cg-*Dock7^m^*+/+*Lepr^db^*/JNarl strain T2D mice (abbreviation *db/db*) and their non-diabetic littermates (control mice, abbreviation *+/+*) were purchased from the National Laboratory Animal Center in Taiwan (Taipei, Taiwan). Ten mice per group for each time point (16, 24 and 32 weeks) were used in the study and maintained under a 12 h/12 h light/dark cycle with free access to water and food. Blood glucose levels were monitored from tail vein blood samples every two weeks (Roche, Mannheim, Germany). All animal procedures were approved by the Institutional Animal Care and Use Committee of China Medical University (Protocol No: 2016-118; approval date: 26 December 2015). 

### 4.3. Retinal Histopathology

**Figure 6:**
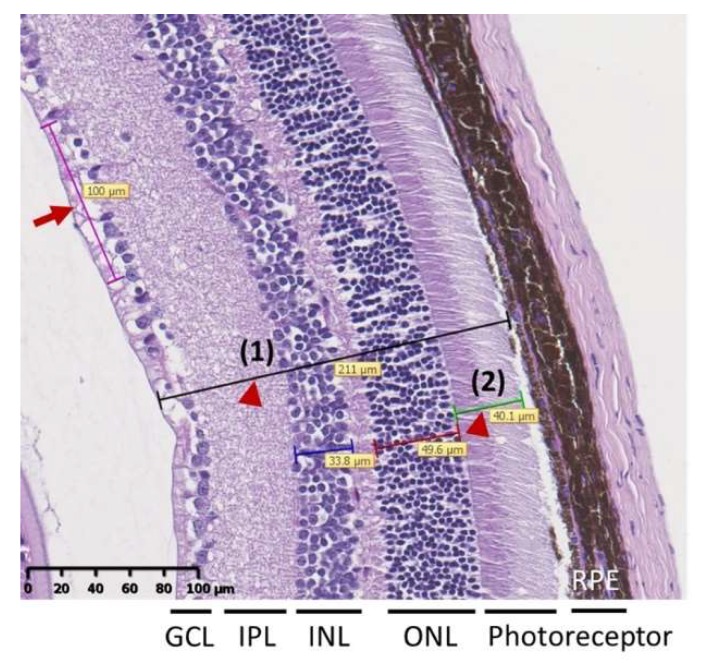
Representative image of retina for measurement of total retina thickness [indicated by arrow head (1)] and thickness of photoreceptor layer [(indicated by arrow head (2)]. Cell number per 100 μm of the GCL was counted based on the linear cell density (arrow). Data were presented as mean ± SD and were measured three times in each hemisphere.

Mice eye balls were fixed in 4% neutral buffered formalin and embedded in paraffin and sectioned along the eye axis (front to back). Sections were subjected to hematoxylin and eosin stain and viewed using a light microscope (Leica DM1000 LED, Wetzlar, Germany) to evaluate the retinal histopathology. Total retinal thickness and the number of cells in the ganglion cell layer (GCL) were used to evaluate the retinal structural abnormality [27,29]. Briefly, after scanning whole tissue slide images by NanoZoomer HT system (Hamamatsu Photonics K.K., Hamamatsu City, Japan), the thickness of the total retina (i.e., between the inner limiting membrane and outer segment photoreceptor) and the thickness of the photoreceptor inner and outer nuclear layers were measured three times in each hemisphere (indicated by arrow heads in Figure 6). Cell number per 100 μm of the GCL was counted based on the linear cell density (indicated by arrow in Figure 6). Each hemisphere was measured three times at adjacent locations. Total retinal thickness and the cell numbers in the GCL were measured by NDP.view2 Viewing software (Hamamatsu Photonics K.K., Hamamatsu City, Japan).

### 4.4. Western Blot

Proteins were extracted from HRECs or mouse retina tissues using radioimmunoprecipitation assay buffer (Sigma Aldrich, St. Louis, MI, USA) containing protease inhibitors and phosphatase inhibitors (Roche, Indianapolis, IN, USA). Protein extracts (20 μg) were electrophoresed on a 12% (*w*/*v*) SDS-polyacrylamide gel and then transferred onto nitrocellulose transfer membranes (Millipore, Billerica, MA, USA) with pore size = 0.2 μm. Membranes were blocked with blocking buffer (Sigma Aldrich, St. Louis, MI, USA) and incubated with primary antibodies (Abs) overnight at 4 °C followed by incubation with horseradish peroxidase conjugated secondary antibody (GeneTex, TX, USA) at room temperature for 1 h. Primary Abs used in the study were RGC-32 (dilution 1:200, Santa Cruz Biotechnology, TX, USA), BCL2-associated X (Bax) (dilution 1:100, Thermo Scientific, Cheshire, UK), B-cell lymphoma 2 (Bcl-2) (dilution 1:1000, Cell Signaling Technology, Danvers, MA, USA), and cleaved caspase-3 (dilution 1:500, Cell Signaling Technology, MA, USA). Anti-β-actin (dilution 1:5000, Novus Biological, Centennial, CO, USA) was used as an internal control. Protein expression was detected with an enhanced chemiluminescence system (Syngene’s ChemiGenius XE Bio Imaging System, Frederick, MD, USA). Quantification analysis was performed using the ImageJ program and normalized to internal control. 

### 4.5. Immunohistochemistry

Paraffin embedded mice eye tissues were sliced into 5-μm sections, deparaffinized, and soaked in a 3% hydrogen peroxide solution in distilled water for 5 min to counteract endogenous peroxidase reactions. Antibody against RGC-32 (dilution 1:100, Santa Cruz Biotechnology, Dallas, TX, USA) was applied to tissue-sections followed by incubation with horseradish peroxidase-conjugated secondary antibody. The presence of peroxidase was revealed by the addition of 3,3′-diaminobenzidine tetrahydrochloride solution and counterstained with hematoxylin. 

### 4.6. Statistical Analysis

Statistical analysis was performed using IBM SPSS Statistics version 22 (IBM Co., Armonk, NY, USA). Results are presented as mean ± SD and differences between means were compared by Student’s *t*-test or one-way analysis of variance (ANOVA) followed by Tukey’s post hoc test as specified in the figure legends. Mann-Whitney U test or Kruskal-Wallis test followed by Dunn’s test was applied to compare the difference when the distribution of data did not follow the normality assumption (Shapiro-Wilk test). *p* < 0.05 was considered statistically significant. 

## Abbreviations

Bax	BCL2-associated X
Bcl-2	B-cell lymphoma 2
DR	diabetic retinopathy
GCL	ganglion cell layer
HREC	human retinal microvascular endothelial cells
INL	inner nuclear layer
IPL	inner plexiform layer
ONL	outer nuclear layers
RGC-32	response gene to complements 32
RPE	retinal pigment epithelium
T2D	type 2 diabetes
VEGF	vascular endothelial growth factor
